# Review of "*The Biology of Echinostomes. From the Molecule to the Community*" by Bernard Fried and Rafael Toledo (eds.)

**DOI:** 10.1186/1756-3305-2-52

**Published:** 2009-11-03

**Authors:** Aneta Kostadinova

**Affiliations:** 1 Institute of Parasitology, Biology Centre of the Academy of Sciences of the Czech Republic, Branišovská 31, 370 05 České Budějovice, Czech Republic; 2 Central Laboratory of General Ecology, Bulgarian Academy of Sciences, 2 Gagarin Street, 1113 Sofia, Bulgaria

## Abstract

Book review of "The Biology of Echinostomes. From the Molecule to the Community" by Bernard Fried and Rafael Toledo (eds.)

## Review

In spite of their limited medical, veterinary or wildlife importance "echinostomes" (a term used in the past for species of *Echinostoma *and recently adopted by some authors for any member of the family Echinostomatidae) may appear to be the most intensely reviewed/overviewed group of trematodes, owing to the enthusiasm of Fried and aficionados and the fact that some species of *Echinostoma *have been used as model organisms in some areas of trematode research. This multi-authored monograph is the second book within the last decade that focuses on "echinostomes" as experimental models. It provides a detailed review of the literature, mostly for the period 1998-2007, thus supplementing "Echinostomes as experimental models for biological research" (Kluwer, Dordrecht) co-edited by Fried & Graczyk in 2000. The present book comprises 13 review papers covering a diversity of topics loosely held together under the theme of "echinostome" and "echinostome-like" (a poorly chosen term for the psilostomid *Ribeiroia ondatrae*) digenean biology and host-parasite interactions. The editors have invited 27 scientists from seven countries (11 from the USA, four from Spain, three each from France and Switzerland, two each from Brazil and the UK and one each from China and Korea). The authorship represents different levels of expertise including authorities in research on *Echinostoma *spp. experimental systems and novices (lead authors with one to four first-authored papers on echinostomatids).

All chapters follow a standard format with helpful subtitles and (usually) conclusions and, in spite of some repetitions/overlaps and lack of data cross-check between some chapters, the evenness of the text appearance indicates substantial editorial effort. The monograph starts with outlines of recent developments in echinostomatid systematics and life-cycles (Esteban & Muñoz-Antoli, Chapter 1) followed by in depth reviews of the complex interactions in the first intermediate snail host (*Biomphalaria*-*Echinostoma *system; Coustau et al., Chapter 2); host-parasite dynamics in the second intermediate hosts (Keeler & Huffman, Chapter 3); and establishment and development within the definitive hosts (Toledo, Chapter 4). Chapter 5 (Fried & Peoples) adds information to the previous book on maintenance of life-cycles in laboratory and tabulates studies carried out in Bernard Fried's laboratory from 1999 to 2007. The title of Chapter 6 "Echinostomes in the wild" (Maldonado & Lanfredi) is misleading since it briefly touches the subject of wildlife infections with *Echinostoma *spp. and surprisingly provides an incomplete listing of studies under the subheading "Echinostomes as experimental models". The text appears speculative in many places e.g. the increasing significance of the "echinostomes" in "emerging and re-emerging zoonoses due to human activities including a rapid increase of the human population, migration, and behavioural changes ..." and the impact of parasitism on wildlife conservation and contains errors of fact e.g. the number of genera of Echinostomatidae currently (actually ever) considered valid. Particularly depressing are Tables 6.3, 6.4, 6.5 and 6.6 due to the many erroneous host Latin names e.g. (this is the correct spelling) *platyrhynchos*, *Turdus*, *Larus*, *albifrons*, *fabalis*, *gentilis*, *Athene*, *peregra*, *Misgurnus anguillicaudatus, Barbatula, Corbicularia, Biomphalaria, Helisoma*) and ... New Zealand is definitely not in Europe! The following chapter (Chai, Chapter 7) lists available data on hosts and distribution (predominantly in South-East Asia) for the 20 human-infecting echinostomatid species as well as data on pathology and immunology in animal host-parasite systems, the latter being a subject of a separate review (Toledo, Chapter 8) which covers the relevant papers on the immunology, pathology and immunodiagnosis of *Echinostoma *spp. in their definitive host with an emphasis on experimental models. Chapter 9 (Marcilla) explores "echinostome" genomics and proteomics, much of the research in the latter field being focussed on the excretory/secretory proteins and their role in host/parasite relationships, high-lights the problems of working with a non-genome verified organism, and provides a useful outline of proteomic methods. Chapter 10 (Sherma & Fried) describes the methods (chromatography and atomic spectrometric approaches) used for the qualitative and quantitative analysis of organic and inorganic constituents of *Echinostoma *spp. (mostly *Echinostoma caproni*) and occasionally host tissues. Chapter 11 (Johnson & McKenzie) is a captivating well-organised review of host-parasite interactions in nature associated with the emergence of *Ribeiroia ondatrae *and echinostomatid infections in the amphibian populations in North America. Chapter 12 (Noland & Graczyk) reviews host-parasite interactions in experimental concurrent infections with *Echinostoma *spp. and other helminths and protozoans and Chapter 13 (Saric et al.) presents brief summary of the use of *Echinostoma *spp. in studies on chemotherapy and explores comprehensively metabolic profiling using an *E. caproni*-mouse model.

The production quality of the monograph is generally good with a few errors of fact, e.g. the "unknown" second intermediate hosts of *Himasthla elongata *in Table 3.1 (both the first and second intermediate hosts of this species are listed together with two references in Table 5.3 in a following chapter). A missing subtitle in Table 11.1 results in all bird definitive hosts listed under 'Reptiles' and Figure 11.6 is missing (replaced by Figure 11.7 which is printed twice). Other than Chapter 6 there are a few misspellings e.g. *Neoacanthoparyphium *(pp. 4, 7), *Vivipara *(p. 70), *Ameiurus melas *(p. 72), *malayanum *(p. 215), *boschas *(p. 255), *Phalacrocorax *(p. 256).

Even with the criticisms mentioned above this compendium of comprehensively reviewed literature (each chapter referenced separately) contains a wealth of information predominantly on *Echinostoma *spp. and thus represents a valuable resource. The monograph will unquestionably be consulted by the aficionados and perhaps broader audience but I doubt it would inspire established researchers to initiate use of *Echinostoma *spp. systems in their laboratories.

## Competing interests

The author declares that they have no competing interests.

